# Genetic Variation in *PSCA* and Risk of Gastric Advanced Preneoplastic Lesions and Cancer in Relation to *Helicobacter pylori* Infection

**DOI:** 10.1371/journal.pone.0073100

**Published:** 2013-09-04

**Authors:** Cosmeri Rizzato, Ikuko Kato, Martyn Plummer, Nubia Muñoz, Federico Canzian

**Affiliations:** 1 German Cancer Research Center (DKFZ), Heidelberg, Germany; 2 Wayne State University, Departments of Oncology and Pathology, Detroit, Michigan, United States of America; 3 International Agency for Research on Cancer (IARC), Lyon, France; 4 National Cancer Institute of Colombia, Bogotá, Colombia; National Cancer Institute, National Institutes of Health, United States of America

## Abstract

SNPs in the Prostate Stem Cell Antigen (*PSCA*) gene have been found associated with gastric cancer (GC) risk in a genome-wide association study. This association has been replicated in several populations. In this study we assessed the impact of *PSCA* genotype on the risk of advanced gastric precancerous lesions and GC. We used baseline gastric histopathology data and DNA from frozen gastric biopsies of 2045 subjects enrolled in a chemoprevention trial for gastric precancerous lesions in Venezuela, and 180 cases of GC from the same area. We analyzed 3 SNPs in the *PSCA* gene (rs2294008, rs9297976 and rs12155758) which were previously found to be associated with GC risk in Europeans. The T allele of rs2294008 was found to be associated with a higher prevalence of atrophic gastritis (OR = 1.44; 95% CI 1.03–2.01 for the dominant model) and intestinal metaplasia (OR = 1.50; 95% CI 1.13–1.98 for the dominant model). We also confirmed the association with higher risk of gastric cancer (OR = 2.34; 95% CI 1.36–4.01 for the allele carriers). SNP rs12155758 was not associated with risk of gastric preneoplastic lesions, but we confirmed its association with higher GC risk (OR 1.95; 95% CI 1.29–2.97 for dominant model). We tested the relevance of the presence of the *Helicobacter pylori cagA* gene, which is known to increase the risk of more severe gastric lesions, but we did not find any clearcut interaction with *PSCA* SNPs in defining risk of gastric precancerous lesions or cancer.

## Introduction

The Prostate Stem Cell Antigen (*PSCA*) gene is located on chromosome 8q24.2 and encodes a 123 amino acid cell surface protein with 30% homology to stem cell antigen type 2 (SCA-2), an immature lymphocyte cell surface marker [1]. *PSCA* is known to be expressed mainly in differentiating cells rather than stem cells [2], [3]. Members of the Thy-1/Ly-6 family, to which PSCA belongs, show a remarkable functional diversity ranging from T-cell activation to apoptosis regulation in the nervous system [4].

PSCA is expressed in the epithelium of several organs, such as prostate, bladder, gallbladder and stomach. Its expression is specific to some populations of cells in the epithelia of these organs: in the gastric epithelium, the main expression site is the isthmus and neck regions, which contain differentiating cells. In particular the expression of PSCA is downregulated in the gastric tissue with intestinal metaplasia [2].

The first genome-wide association study (GWAS) on gastric cancer (GC), performed in a Japanese population, indentified an association between SNP rs2294008 () of *PSCA* and risk of the diffuse type of GC. Substitution of the C allele with the risk allele T at rs2294008 in the first exon creates a novel translation start site (Met instead of Thr), thereby extending the protein by 9 amino acids and affecting transcriptional activity of the gene [2]. The association between rs2294008 and GC risk has been replicated in other Asian and Caucasian populations [5]–[9]. An association with intestinal type GC was also found both in Caucasians and Asians, albeit with smaller odds ratios than with diffuse type [5], [6], [10], [11].

Two other SNPs were previously found to be associated with GC in Europeans [6]. These SNPs are rs9297976, located at 9.6 kb upstream of *PSCA* and 0.8 kb upstream of *JRK*; and rs12155758, located at 1.7 kb downstream of *PSCA*.

Here, we conducted a study to assess the impact of these 3 *PSCA* SNPs on the risk of advanced gastric precancerous lesions and gastric cancer for the first time in a Latin American (Venezuelan) population. This population is at relatively high risk of gastric cancer (the incidence rate is of 10.4 new cases per 100,000 persons per year and the mortality is of 8.9 deaths per 100,000 persons per year (http://globocan.iarc.fr/), in particular the states of Tachira (where the samples were collected) shows a gastric cancer mortality rate 3–4 times higher than the remainder of the country [12]. The Venezuelan population has also very high rates of infection with *Helicobacter pylori (H. pylori)*, the best known risk factor for gastric cancer [13], [14].

We also tested the relevance of the presence of the *H. pylori cagA* gene, which in a previous study [15] we have demonstrated to strongly increase the risk of more advanced lesions. The *cagA* gene resides within the cytotoxin-associated gene pathogenicity island (cagPAI), the best characterized *H. pylori* virulence marker; this region forms a type IV secretion system that translocates bacterial products into the host cell. cagA is responsible for most of the *H. pylori* associated malignant phenotypes: it triggers interleukin-8 secretion, priming an inflammatory response, promotes cell proliferation, scattering and migration [16], [17]. The prevalence of *cagA*-positive strains in Venezuelan populations has been estimated between 59% and 95% in *H. pylori*-positive patients [18], [19].

## Materials and Methods

### Ethics Statement

All participants of the prevention trial signed an informed written consent, while for the participant to the case control study oral informed consent was obtained according to the normal procedure implemented at the Cancer Control Center and at the hospital in San Cristobal, where the samples were collected, at the time the study took place.

Both studies were approved by the ethical review boards of the International Agency for Research on Cancer (IARC) Ethical Committee in Lyon, France, and the Cancer Control Center in San Cristobal, Venezuela.

### Study Population

The randomized trial that provided the basis for this study has been described previously [15]. Eligible subjects were participants in the GC control program of Tachira State, Venezuela, between 35 and 69 years of age. All subjects underwent gastroscopic examination with collection of gastric biopsies, blood, and urine specimens, and they were administered a questionnaire on sociodemographic and lifestyle variables by a trained interviewer. During the study recruitment period from July 1991 to February 1995, there were 4349 eligible subjects, of whom 2272 were invited to participate in the trial. Of these, 72 refused to participate. For 155 subjects the DNA from biopsies was not available anymore or the quality was insufficient for genotyping, leaving thus a total of 2045 subjects who were included in statistical analyses.

Gastric cancer cases were identified in the general hospital at San Cristobal, capital of Tachira State, between January 1991 and January 1997 as part of a case control study running in parallel with the intervention trial [13], [20]. In order to be eligible for inclusion in the study, cases had to be resident in Tachira for at least 5 years, be over 35 years old, have histologically confirmed gastric cancer and not have had previous gastric surgery. Non-epithelial tumours of the stomach were excluded. Cases were classified according to the Lauren classification. Five of the 7 biopsies taken from each subject at baseline were used for histopathological assessment. These were taken from the antrum (3 biopsies), the incisura anguli (1 biopsy) and the corpus (1 biopsy). Biopsies were fixed in buffered formalin and stained with hematoxylin–eosin and Giemsa. One of the 3 pathologists at the Cancer Control Center classified each of the 5 biopsies as indicative of normal mucosa, superficial gastritis, chronic gastritis, chronic atrophic gastritis, intestinal metaplasia or dysplasia, as previously described [13], [20] Controls from the case control study are not included in the present analysis as they did not provide gastric biopsies and so do not have comparable data on *H. pylori* infection.

### SNPs Selection and Genotyping

We selected the three *PSCA* SNPs that were previously reported to be associated with GC risk in Europeans [6]. We did not type SNP rs2976392, originally found associated with gastric cancer risk in the first GWAS on gastric cancer [2], because it is in very high or complete linkage disequilibrium with rs2294008 (0.97≤r^2^≤1.0) in 6 populations with origin from Europe, North or South America analyzed in the 1000 genomes project (http://www.1000genomes.org) [21] and therefore likely in the Venezuelan population as well.

Total DNA was extracted from gastric biopsy specimens after digestion with proteinase K. Briefly, biopsies were incubated in 250 µL of a solution of 10 mM Tris – HCl (pH 8.0), 5 mM EDTA, 0.1% sodium dodecyl sulfate, and 0.1 mg/mL Proteinase K for at least 2 hours at 55°C. Proteinase K was inactivated by incubation at 95°C for 10 minutes.

Genotyping was performed using an allele-specific PCR-based KASPar SNP genotyping system (KBiosciences, Hoddesdon, UK). Thermocycling was performed according to the manufacturer’s instructions. Detection was performed using an ABI PRISM 7900 HT sequence detection system with SDS 2.2 software (Applied Biosystems, Foster City, CA, USA).

The presence of the *H. pylori cagA* gene in gastric biopsies was previously assessed by reverse hybridization using a line probe assay or a DNA enzyme immunoassay at Delft Diagnostic Laboratory [22], [23]. In addition, we typed two SNPs in the *cagA* gene in position 154 (cagA154_GA) and 858 (cagA858_CT), by allele-specific PCR-based KASPar SNP genotyping system. The presence of the two polymorphic sites has been assessed by sequencing in a small subset of the same population [24]. Furthermore, the results obtained with this assay were compared with the sequencing results with 100% concordance. A sample was defined as cagA positive when it showed a signal in at least two out of three PCRs (i.e. the reverse hybridization/DNA enzyme immunoassay and the two SNP assays).

### Statistical Analysis

The response variable in this study was global histological diagnosis, which was divided into 5 groups: gastric cancer; dysplasia; IM; atrophic gastritis; and normal epithelium to chronic gastritis. The last group served as the control group in this study because the combined frequency of normal epithelium and superficial gastritis in this population was less than 5%. Multinominal logistic regression analysis was employed, using the SAS CATMOD procedure, to estimate odds ratios (ORs) and 95% confidence intervals (CIs) associated with SNPs for atrophic gastritis, IM, dysplasia and gastric cancer, in comparison with controls. All ORs were adjusted for basic demographic variables (gender, age and educational level), and other risk factors reported previously in this population (cigarette smoking, and duration of refrigerator use) [25]. The equality of ORs between strata (H: β1−β2 = 0) was tested using a one degree of freedom Wald chi-square statistic. In the dominant model, heterozygotes and homozygotes for the minor allele were considered a single group of minor allele carriers. In order to take into account the large number of tests we used the Bonferroni correction to set the significance threshold, resulting in a final threshold of p<0.004 (if we divide 0.05/12 (3 polymorphisms*4 diagnostic endpoints)).

## Results

Basic characteristics of the population included in this study are presented in Table 1. Genotype success was >95%. Blinded duplicate samples (16.7%) included for quality control showed >99% genotype concordance. The genotype frequencies for all SNPs in controls were in accordance with Hardy–Weinberg equilibrium (data not shown).

**Table 1:** Characteristics of study population.

		Pathological diagnoses			
Characteristics	Normal/non-atrophicgastritis	Atrophic gastritis	Intestinal metaplasia	Dysplasia	Gastric cancer
Age					
= <39	299 (28.2%)	73 (22.9%)	92 (10.8%)	12 (10.8%)	11 (6.1%)
40–49	423 (39.9%)	113 (35.4%)	169 (30.5%)	38 (34.2%)	26 (14.4%)
50–59	233 (22.0%)	91 (28.5%)	175 (31.6%)	29 (26.1%)	42 (23.3%)
> = 60	106 (10.0%)	42 (13.2%)	118 (21.3%)	32 (28.8%)	101 (56.1%)
Gender					
Female	575 (54.2%)	174 (54.6%)	277 (50.0%)	57 (51.4%)	53 (29.4%)
Male	486 (45.8%)	145 (45.5%)	277 (50.0%)	54 (48.7%)	127 (70.6%)
H. pylori (HP) status					
No HP	111 (10.5%)	27 (8.5%)	37 (6.7%)	5 (4.5%)	7 (3.9%)
cagA-negative HP	387 (36.5%)	76 (23.8%)	82 (14.8%)	14 (12.6%)	45 (25.0%)
cagA-positive HP	563 (53.1%)	216 (67.7%)	435 (78.5%)	92 (82.9%)	128 (71.1%)
Cancer subtypes					
Intestinal	–	–	–	–	103 (57.2%)
Diffuse/other	–	–	–	–	77 (42.8%)
Non-cardia	–	–	–	–	162 (90.0%)
Cardia	–	–	–	–	18 (10.0%)
Total	1061	319	554	111	180

We assessed the risk for gastric precancerous lesions and gastric cancer, in comparisons with normal and non-atrophic gastritis, according to the genotypes in *PSCA* SNPs (table 2).

**Table 2:** ORs for precancerous lesions and GC, in comparison with normal or non-atrophic gastritis, according to individual *PSCA* SNPs.

Histological diagnoses	No.	OR	No.	OR	(95% CI)	No.	OR	(95% CI)	P_trend_	OR	(95% CI)
rs12155758											
	GG		AG			AA				Dominant	
Normal/non-atrophic gastritis	409	–	491	–		156	–		–	–	
Atrophic gastritis	115	1	152	1.12	(0.84–1.48)	48	1.09	(0.74–1.61)	0.526	1.11	(0.85–1.45)
Intestinal metaplasia	205	1	257	1.09	(0.86–1.39)	85	1.08	(0.77–1.50)	0.538	1.09	(0.87–1.36)
Dysplasia	43	1	48	0.97	(0.62–1.52)	19	1.15	(0.64–2.08)	0.726	1.02	(0.67–1.54)
Gastric cancer	46	1	105	**2.13**	**(1.38–3.28)**	25	1.46	(0.80–2.67)	**0.041**	**1.95**	**(1.29–2.97)**
**rs2294008**											
	**CC**		**CT**			**TT**				**Dominant**	
Normal/non-atrophic gastritis	231	–	507	–		319	–		–	–	
Atrophic gastritis	53	1	167	*1.49*	*(1.05–2.11)*	98	1.36	(0.93–1.99)	0.181	*1.44*	*(1.03–2.01)*
Intestinal metaplasia	93	1	291	**1.56**	**(1.16–2.09)**	169	*1.40*	*(1.02–1.93)*	0.084	*1.50*	*(1.13–1.98)*
Dysplasia	17	1	56	1.68	(0.94–3.00)	37	1.71	(0.92–3.16)	0.114	1.69	(0.97–2.93)
Gastric cancer	23	1	86	**2.16**	**(1.23–3.82)**	69	**2.6**	**(1.44–4.68)**	**0.002**	**2.34**	**(1.36–4.01)**
**rs9297976**											
	**TT**		**CT**			**CC**				**Dominant**	
Normal/non-atrophic gastritis	417	–	485	–		147	–		–	–	
Atrophic gastritis	127	1	149	1.05	(0.80–1.38)	37	0.85	(0.56–1.29)	0.649	1	(0.77–1.30)
Intestinal metaplasia	219	1	267	1.12	(0.89–1.42)	60	0.77	(0.54–1.11)	0.495	1.04	(0.83–1.30)
Dysplasia	45	1	56	1.19	(0.77–1.82)	10	0.62	(0.30–1.28)	0.497	1.05	(0.69–1.58)
Gastric cancer	85	1	72	0.86	(0.58–1.28)	20	0.6	(0.32–1.12)	0.109	0.8	(0.55–1.16)

Odds ratios were adjusted for age, gender, smoking status, length of refrigerator use, educational level. Values in bold are statistically significant (p<0.004), in italic p-value<0.017 (p = 0.05/3 polymorphisms).

For rs2294008, we observed an increased risk of atrophic gastritis (OR = 1.49; 95% CI 1.05–2.11 in heterozygous carriers and OR = 1.44; 95% CI 1.03–2.01 in the dominant model), and IM (OR = 1.56; 95% CI 1.16–2.09 in heterozygous carriers; OR = 1.40; 95% CI 1.02–1.93 in homozygous carriers and OR = 1.50; 95% CI 1.13–1.98 in the dominant model). We also confirmed the increased risk for overall gastric cancer in both heterozygous and homozygous carriers of T allele of rs2294008 (respectively OR = 2.16; 95% CI 1.23–3.82; OR = 2.60; 95% CI 1.44–4.68; P_trend_ = 0.002) and a significant association with the T allele under the dominant model (OR = 2.34; 95% CI 1.36–4.01).

For rs12155758 we did not observe any statistically significant association with gastric preneoplastic lesions, nevertheless heterozygous carriers of the A allele were associated with increased risk of GC (OR 2.13; 95% CI 1.38–3.28) and under the dominant model the A allele was also associated with increased GC risk (OR = 1.95; 95% CI 1.29–2.97).

rs9297976 did not show any statistically significant association with preneoplastic lesion or GC risk.

### Associations with GC Subgroups

We performed a sub-group analysis in subjects of the significant associations with gastric cancer in table 2 to see if the strength of the association differed by histological type (103 cases of intestinal subtypes, 56 diffuse and 21 other types) and anatomical sub-site (18 cases of cardia and 162 not cardia cases). The results are shown in table 3; rs9297976 is omitted because it showed no association with gastric cancer. None of the observed differences of association between GC subgroups was statistically significant (table 3).

**Table 3:** ORs for gastric cancer cases stratified by histology and anatomic localization, in comparison with normal or non-atrophic gastritis, according to individual *PSCA* SNPs.

rs12155758														
	GG		AG				AA					Dominant		
Histological diagnoses	No.	OR	No.	OR	(95% CI)	P-value for difference	No.	OR	(95% CI)	P-value for difference	P-values for trend	OR	(95% CI)	P-value for difference
Normal/non-atrophic gastritis	409	–	491	–			156	–			–	–		
Intestinal type	28	1	58	*1.95*	*(1.14–3.32)*	0.624	14	1.36	(0.64–2.88)	0.766	0.154	1.76	(1.07–3.00)	0.587
Diffuse and other type	18	1	47	**2.38**	**(1.32–4.30)**		11	1.61	(0.71–3.65)		0.073	*2.18*	*(1.23–3.88)*	
Non-cardia cancer	42	1	94	**2.08**	**(1.33–3.23)**	0.626	22	1.41	(0.76–2.62)	0.618	0.06	**1.90**	**(1.24–2.92)**	0.586
Cardia cancer	4	1	11	2.87	(0.85–9.65)		3	2.18	(0.44–10.77)		0.201	2.69	(0.83–8.73)	
**rs2294008**														
	**CC**		**CT**				**TT**					**Dominant**		
Normal/non-atrophic gastritis	231	–	507	–			319	–			–	–		
Intestinal type	14	1	50	2.12	(1.05–4.27)	0.918	37	2.33	(1.12–4.84)	0.666	0.033	2.21	(1.13–4.30)	0.791
Diffuse and other type	9	1	36	2.24	(1.02–4.91)		32	*2.96*	*(1.33–6.61)*		0.007	*2.53*	*(1.20–5.35)*	
Non-cardia cancer	21	1	75	*2.06*	*(1.15–3.69)*	0.500	64	**2.61**	**(1.43–4.76)**	0.933	0.002	**2.28**	**(1.32–3.96)**	0.690
Cardia cancer	2	1	11	3.72	(0.74–18.65)		5	2.41	(0.42–13.92)		0.459	3.20	(0.66–15.41)	

Odds ratios were adjusted for age, gender, smoking status, length of refrigerator use, educational level. Values in bold are statistically significant (p<0.004), in italic p-value<0.017 (p = 0.05/3 polymorphisms).

### Associations by cagA Status

We also conducted a sub-group analysis of significant associations with GC and with precancerous lesions under the dominant model, to see if the strength of association differed by *cagA* status (Table 4). Associations that were not significant in table 2 are omitted. We also combined intestinal metaplasia and dysplasia into a single category due to the small number of dysplasias in the cagA negative group [15].

**Table 4:** ORs for pre-neoplastic lesions and gastric cancer cases stratified by diagnosis and *cagA* status, in comparison with normal or non-atrophic gastritis, according to individual *PSCA* SNPs.

		Normal/non-atrophic gastritis	Atrophic gastritis			Intestinal metaplasia+Dysplasia			Cancer		
Genotypes	*cagA*	No.	No	OR	(95% CI)	No.	OR	(95% CI)	No.	OR	(95% CI)
**rs12155758**											
GG	+	231	80	1.00		189	1.00		31	1.00	
AG/AA		329	133	1.20	(0.84–1.61)	332	1.20	(0.96–1.60)	95	**2.20**	**(1.34–3.68)**
GG	–	178	35	1.00		59	1.00		15	1.00	
AG/AA		318	67	1.10	(0.70–1.74)	77	0.77	(0.52–1.15)	35	1.69	(0.78–3.63)
P-values for interaction between SNP and *cagA* status					0.818			**0.046**			0.443
**rs2294008**											
CC	+	136	40	1.00		82	1.00		16	1.00	
CT/TT		427	176	1.40	(0.94–2.09)	443	**1.80**	**(1.29–2.42)**	112	*2.40*	*(1.25–4.43)*
CC	–	95	13	1.00		28	1.00		7	1.00	
CT/TT		399	89	1.70	(0.88–3.18)	110	1.03	(0.63–1.68)	43	2.55	(0.90–7.27)
P-values for interaction between SNP and *cagA* status					0.720			0.056			0.986

Odds ratios were adjusted for age, gender, smoking status, length of refrigerator use, educational level. *cagA* negative includes subjects who were not infected by *H. pylori* (187 subjects)and subjects who were infected by *H. pylori* but carried a *cagA*-negative strain (604 subjects). Values in bold are statistically significant (p<0.004), in italic p-value<0.017 (p = 0.05/3 polymorphisms).

A borderline significant interaction between *H. pylori cagA* status and genotypes at rs2294008 was observed for the combined group of subjects with IM or dysplasia (p =  P = 0.056), in which the association appeared stronger in the *cagA*-positive group (OR = 1.80, 95% CI 1.29–2.42) than the *cagA*-negative group (OR = 1.03; 95% CI 0.63–1.68). However, no such interaction was observed for GC, where the ORs appeared equally strong in both sub-groups (OR = 2.40 vs OR = 2.55, p = 0.99) (Table 4).

## Discussion

We report here the re-evaluation of 3 SNPs in *PSCA* found to be associated with gastric cancer risk in Europeans [5], [6] and their possible involvement in risk of precancerous and cancerous lesions in relation with the infection with *cagA*-positive *H. pylori*.

Three previous GWAS found a significant association of the T allele of rs2294008, a functional SNP in the *PSCA* gene, with the risk of gastric and bladder cancers [2], [26], [27] and the C allele for duodenal ulcer [28]. Several case-control studies confirmed the association of this SNP with GC in Asian and Caucasian populations. Meta-analyses [10], [11], [29], [30] showed ORs ranging between 1.41–1.66 for the dominant model and between 1.14–1.33 for the recessive model. The two other *PSCA* SNPs of our study were previously found to be associated with GC risk in Europeans [6]. We confirm the association between the T allele of rs2294008 and A allele of rs12155758 with GC risk.

Atrophy, intestinal metaplasia, dysplasia, and cancer develop sequentially over several decades in susceptible patients with persistent *H. pylori*-associated gastritis [31]. Lochhead and colleagues [5] found that the rs2294008 T allele is associated also with atrophic gastritis. The results of the present study shows the association between the T allele of rs2294008 with risk of atrophic gastritis and IM. A greater degree of the association is observed also for dysplasia, although it is not statistically significant, possibly due to the small number of samples analyzed. We observe a gradient of risk consistent with the progression from less severe to more severe preneoplastic lesions and with cancer. These results should be interpreted with caution due to the small size of the individual groups of subjects with specific preneoplastic lesions. More studies, with larger sample sizes are needed to draw definitive conclusions on this point.

*In vitro* reporter assays showed that the T allele of rs2294008 reduced the transcriptional activity of the *PSCA* promoter in both gastric and bladder cell lines [2], [27]. Furthermore a recent functional study showed that the genetic variation in *PSCA* could alter subcellular localization and stability of the protein [28]. These findings led to the hypothesis that susceptibility to duodenal ulcer and gastric cancer is influenced by genetic variation in *PSCA* through a growth-promoting effect of the T allele and an effect on T-cell activation by the C allele [28].

Intestinal and diffuse type gastric adenocarcinomas are different in their epidemiological features, etiology and prognosis [32]–[36]. Consistently with studies in Europeans and meta-analyses of studies in Asians [5], [11], [29], [30], we observe an association of this SNP with all histological types of GC, without any statistically significant evidence for difference in risk between GC subtypes according to histology or location. It should be noticed however that the sample size of the subgroups in this study is rather small.

The association between rs2294008 and *H. pylori* infection has been investigated in 3 studies, but none of them have found a statistically significant association, except for a weak interaction between the SNP and the infection found in a European population [5], [6], [37]. We explored the interaction between *PSCA* polymorphisms and the presence or absence of *cagA* strains rather than of *H. pylori* infection, because in a previous study [15] on the same population we observed a strong correlation between *cagA* presence and risk of advanced premalignant lesions. We did not observe any convincing interaction between *PSCA* SNPs and *cagA* status for risk of preneoplastic gastric lesions or GC. The weakly significant interaction between rs12155758 and *cagA* status with respect to risk of IM/dysplasia is difficult to interpret because rs12155758 did now show an association with risk of IM/dysplasia on its own. These observations suggest that PSCA may be involved in gastric carcinogenesis through both cagA-dependent and -independent pathways.

We realize that our study has some limitations. First, although the overall study is rather large, the sample size for specific subgroups is rather small. Furthermore, inferring causal relationship for the observed associations in a cross-sectional study might be difficult, because temporal relations between exposures and outcomes are not clear. Yet, the cross-sectional analysis has an advantage in accumulating histological changes developing over several decades as *H. pylori* is generally acquired in the childhood in high-risk populations [38]. Third, we do not have information regarding the ethnicity of study subjects. There are differences in the allelic distributions of these SNPs in different populations (see supplementary table S1), however the frequencies we observed are similar to the other Latin American populations for which there are data available (supplementary table S1). Although we cannot formally demonstrate that these three variants adequately cover those tagged in the European population studied by Sala *et al*. [6], the Venezuelan population we have studied has a strong component of European ancestry, therefore the linkage disequilibrium pattern should arguably be not very different. Finally, our study was limited to a Venezuelan population and the results cannot necessarily be extrapolated to other populations.

In summary we showed that a functional SNP in the *PSCA* gene is associated with risk of advanced precancerous gastric lesions and GC and the assessment of the risk seems not to be modified by the presence or absence of *H.pylori* strains carrying the gene encoding the bacterial cytotoxin cagA.

## Supporting Information

Table S1 **Allele and genotype frequencies of the studied Venezuelan population and the populations from the 1000 Genomes Project.**
(DOCX)
