# Supplementary material for: Genetic Variation in PSCA and Risk of Gastric Advanced Preneoplastic Lesions and Cancer in Relation to Helicobacter pylori Infection
Source: PLoS One. 2013 Sep 4;8(9):e73100. doi: 10.1371/journal.pone.0073100 (PMC3762831; doi:10.1371/journal.pone.0073100)
Supplement: Table S1 — Allele and genotype frequencies of the studied Venezuelan population and the populations from the 1000 Genomes Project. (DOCX) [file pone.0073100.s001.docx]

**Table S1.** Allele and genotype frequencies of the studied Venezuelan population and the populations from the 1000 Genomes Project.

| rs2294008 | | | | | |
| --- | --- | --- | --- | --- | --- |
| Population | Alleles C | Alleles T | Genotypes C\|C | Genotypes C\|T | Genotypes T\|T |
| 1000GENOMES:phase_1_ALL | 0.568 | 0.432 | 0.325 | 0.485 | 0.190 |
| 1000GENOMES:phase_1_AFR | 0.622 | 0.378 | 0.382 | 0.480 | 0.138 |
| 1000GENOMES:phase_1_AMR | 0.434 | 0.566 | 0.171 | 0.525 | 0.304 |
| 1000GENOMES:phase_1_ASN | 0.622 | 0.378 | 0.416 | 0.413 | 0.171 |
| 1000GENOMES:phase_1_ASW | 0.500 | 0.500 | 0.246 | 0.508 | 0.246 |
| 1000GENOMES:phase_1_CEU | 0.588 | 0.412 | 0.329 | 0.518 | 0.153 |
| 1000GENOMES:phase_1_CHB | 0.742 | 0.258 | 0.546 | 0.392 | 0.062 |
| 1000GENOMES:phase_1_CHS | 0.725 | 0.275 | 0.540 | 0.370 | 0.090 |
| 1000GENOMES:phase_1_CLM | 0.367 | 0.633 | 0.117 | 0.500 | 0.383 |
| 1000GENOMES:phase_1_EUR | 0.555 | 0.445 | 0.293 | 0.525 | 0.182 |
| 1000GENOMES:phase_1_FIN | 0.468 | 0.532 | 0.215 | 0.505 | 0.280 |
| 1000GENOMES:phase_1_GBR | 0.567 | 0.433 | 0.281 | 0.573 | 0.146 |
| 1000GENOMES:phase_1_IBS | 0.536 | 0.464 | 0.143 | 0.786 | 0.071 |
| 1000GENOMES:phase_1_JPT | 0.376 | 0.624 | 0.135 | 0.483 | 0.382 |
| 1000GENOMES:phase_1_LWK | 0.572 | 0.428 | 0.289 | 0.567 | 0.144 |
| 1000GENOMES:phase_1_MXL | 0.424 | 0.576 | 0.167 | 0.515 | 0.318 |
| 1000GENOMES:phase_1_PUR | 0.518 | 0.482 | 0.236 | 0.564 | 0.200 |
| 1000GENOMES:phase_1_TSI | 0.602 | 0.398 | 0.367 | 0.469 | 0.163 |
| 1000GENOMES:phase_1_YRI | 0.761 | 0.239 | 0.580 | 0.364 | 0.057 |
| Venezuelan | 0.438 | 0.562 | 0.188 | 0.500 | 0.312 |
| rs9297976 | | | | | |
| Population | Alleles C | Alleles T | Genotypes C\|C | Genotypes C\|T | Genotypes T\|T |
| 1000GENOMES:phase_1_ALL | 0.391 | 0.609 | 0.149 | 0.483 | 0.368 |
| 1000GENOMES:phase_1_AFR | 0.447 | 0.553 | 0.224 | 0.447 | 0.329 |
| 1000GENOMES:phase_1_AMR | 0.331 | 0.669 | 0.122 | 0.420 | 0.459 |
| 1000GENOMES:phase_1_ASN | 0.323 | 0.677 | 0.094 | 0.458 | 0.448 |
| 1000GENOMES:phase_1_ASW | 0.328 | 0.672 | 0.082 | 0.492 | 0.426 |
| 1000GENOMES:phase_1_CEU | 0.447 | 0.553 | 0.141 | 0.612 | 0.247 |
| 1000GENOMES:phase_1_CHB | 0.376 | 0.624 | 0.144 | 0.464 | 0.392 |
| 1000GENOMES:phase_1_CHS | 0.385 | 0.615 | 0.120 | 0.530 | 0.350 |
| 1000GENOMES:phase_1_CLM | 0.292 | 0.708 | 0.083 | 0.417 | 0.500 |
| 1000GENOMES:phase_1_EUR | 0.433 | 0.567 | 0.156 | 0.554 | 0.290 |
| 1000GENOMES:phase_1_FIN | 0.382 | 0.618 | 0.129 | 0.505 | 0.366 |
| 1000GENOMES:phase_1_GBR | 0.438 | 0.562 | 0.157 | 0.562 | 0.281 |
| 1000GENOMES:phase_1_IBS | 0.500 | 0.500 | 0.143 | 0.714 | 0.143 |
| 1000GENOMES:phase_1_JPT | 0.197 | 0.803 | 0.011 | 0.371 | 0.618 |
| 1000GENOMES:phase_1_LWK | 0.454 | 0.546 | 0.216 | 0.474 | 0.309 |
| 1000GENOMES:phase_1_MXL | 0.318 | 0.682 | 0.106 | 0.424 | 0.470 |
| 1000GENOMES:phase_1_PUR | 0.391 | 0.609 | 0.182 | 0.418 | 0.400 |
| 1000GENOMES:phase_1_TSI | 0.454 | 0.546 | 0.194 | 0.520 | 0.286 |
| 1000GENOMES:phase_1_YRI | 0.523 | 0.477 | 0.330 | 0.386 | 0.284 |
| Venezuelan | 0.359 | 0.641 | 0.125 | 0.469 | 0.407 |
| rs12155758 | | | | | |
| Population | Alleles A | Alleles G | Genotypes A\|A | Genotypes A\|G | Genotypes G\|G |
| 1000GENOMES:phase_1_ALL | 0.214 | 0.786 | 0.056 | 0.316 | 0.628 |
| 1000GENOMES:phase_1_AFR | 0.083 | 0.917 | 0.012 | 0.142 | 0.846 |
| 1000GENOMES:phase_1_AMR | 0.381 | 0.619 | 0.144 | 0.475 | 0.381 |
| 1000GENOMES:phase_1_ASN | 0.161 | 0.839 | 0.028 | 0.266 | 0.706 |
| 1000GENOMES:phase_1_ASW | 0.139 | 0.861 | 0.033 | 0.213 | 0.754 |
| 1000GENOMES:phase_1_CEU | 0.241 | 0.759 | 0.047 | 0.388 | 0.565 |
| 1000GENOMES:phase_1_CHB | 0.082 | 0.918 |  | 0.165 | 0.835 |
| 1000GENOMES:phase_1_CHS | 0.120 | 0.880 | 0.010 | 0.220 | 0.770 |
| 1000GENOMES:phase_1_CLM | 0.417 | 0.583 | 0.200 | 0.433 | 0.367 |
| 1000GENOMES:phase_1_EUR | 0.259 | 0.741 | 0.063 | 0.391 | 0.546 |
| 1000GENOMES:phase_1_FIN | 0.339 | 0.661 | 0.140 | 0.398 | 0.462 |
| 1000GENOMES:phase_1_GBR | 0.253 | 0.747 | 0.056 | 0.393 | 0.551 |
| 1000GENOMES:phase_1_IBS | 0.321 | 0.679 |  | 0.643 | 0.357 |
| 1000GENOMES:phase_1_JPT | 0.292 | 0.708 | 0.079 | 0.427 | 0.494 |
| 1000GENOMES:phase_1_LWK | 0.093 | 0.907 | 0.010 | 0.165 | 0.825 |
| 1000GENOMES:phase_1_MXL | 0.417 | 0.583 | 0.121 | 0.591 | 0.288 |
| 1000GENOMES:phase_1_PUR | 0.300 | 0.700 | 0.109 | 0.382 | 0.509 |
| 1000GENOMES:phase_1_TSI | 0.194 | 0.806 | 0.020 | 0.347 | 0.633 |
| 1000GENOMES:phase_1_YRI | 0.034 | 0.966 |  | 0.068 | 0.932 |
| Venezuelan | 0.433 | 0.567 | 0.151 | 0.478 | 0.371 |
